# Optimization of the Transcranial Magnetic Stimulation Protocol by Defining a Reliable Estimate for Corticospinal Excitability

**DOI:** 10.1371/journal.pone.0086380

**Published:** 2014-01-24

**Authors:** Koen Cuypers, Herbert Thijs, Raf L. J. Meesen

**Affiliations:** 1 REVAL Rehabilitation Research Centre, Biomedical Research Institute, Faculty of Medicine and Life Sciences, Hasselt University, Diepenbeek, Belgium; 2 Motor Control Laboratory, Research Center for Movement Control and Neuroplasticity, Department of Biomedical Kinesiology, Group Biomedical Sciences, K.U. Leuven, Heverlee, Belgium; 3 I-BioStat, Interuniversity Institute for Biostatistics and statistical Bioinformatics, Hasselt University, Diepenbeek, Belgium; 4 I-BioStat, Interuniversity Institute for Biostatistics and statistical Bioinformatics, Leuven University, Leuven, Belgium; University of Montreal, Canada

## Abstract

The goal of this study was to optimize the transcranial magnetic stimulation (TMS) protocol for acquiring a reliable estimate of corticospinal excitability (CSE) using single-pulse TMS. Moreover, the minimal number of stimuli required to obtain a reliable estimate of CSE was investigated. In addition, the effect of two frequently used stimulation intensities [110% relative to the resting motor threshold (rMT) and 120% rMT] and gender was evaluated. Thirty-six healthy young subjects (18 males and 18 females) participated in a double-blind crossover procedure. They received 2 blocks of 40 consecutive TMS stimuli at either 110% rMT or 120% rMT in a randomized order. Based upon our data, we advise that at least 30 consecutive stimuli are required to obtain the most reliable estimate for CSE. Stimulation intensity and gender had no significant influence on CSE estimation. In addition, our results revealed that for subjects with a higher rMT, fewer consecutive stimuli were required to reach a stable estimate of CSE. The current findings can be used to optimize the design of similar TMS experiments.

## Introduction

Corticospinal (CS) excitability can be estimated by measuring motor evoked potentials (MEPs) elicited by transcranial magnetic stimulation (TMS). MEPs provide information about the functionality of the human nervous system and are commonly measured in both research and clinical settings for diagnostic and therapeutic purposes. Because the MEP amplitude is highly variable within the same subject [1]–[4], consecutive measurements are required to obtain a reliable estimate of the CSE of the stimulated cortical site. Although the nature of MEP amplitude variability remains mainly unclear, randomness in processes such as the firing of neurons and motor neuron transmission might play a role [3], [5]. Additionally, trial-to-trial fluctuations in MEP amplitude might be caused by rapid fluctuating changes in cortical and spinal excitability [1], [6], [7]. It is reported that other physiological factors influence the MEP amplitude as well, such as pre-stimulus contraction [1], [8], attention [9], arousal, desynchronization of action potentials [3], and afferent feedback [10]. Besides these physiological factors, physical parameters such as coil orientation [11], optimal scalp location [12] and environmental noise might also play an important role in the variability of the MEP amplitude.

Whereas, in most studies 6 to 10 pulses were applied to determine CSE of the region of interest [13], it is still unclear how many TMS pulses are required to acquire a reliable estimate of CSE. Maximizing reliability is important for the assessment of neurophysiological effects caused by interventions that affect CSE. Although TMS has already been used for several decades, previous studies showed no consistency regarding the number of pulses used for measuring CSE.

Another relevant question is whether the number of pulses is mediated by factors such as stimulation intensity and/or gender. Previously, a sigmoidal relationship between stimulation intensity and average MEP amplitude was reported [5], showing that well-tolerated stimulation intensities, which are represented in the middle of the recruitment curve are sensitive for detecting changes in CSE as they are not subjected to either floor or ceiling effects. When applying single-pulse TMS at rest, two intensities relative to the resting motor threshold (rMT) are frequently applied to measure CSE, respectively 110% rMT [14]–[16] and 120% rMT [17]–[19]. However, it is not clear whether the number of pulses required to acquire a reliable estimate of CSE is mediated by these different stimulation intensities. Another parameter that might influence CME measurement is gender, as previous findings indicated that MEP variation was more pronounced in females [5], [20], [21]. Nonetheless, it should be mentioned that increased MEP variability was only reported using a paired-pulse TMS paradigm for measuring changes in intracortical inhibition [20], [22]. Yet, it is not clear whether similar effects will be found when single-pulse TMS is used.

The goal of the current study was to optimize the TMS protocol for acquiring a reliable estimate of CSE. Therefore, the first aim of the study was to examine the number of stimuli required to obtain a reliable estimate of CSE. The second aim was to evaluate the effect of two frequently used intensities (110% rMT and 120% rMT) on the reliability of CSE. And finally, we aimed to investigate whether gender acts as a covariate in estimating CSE.

## Materials and Methods

### Ethics Statement

Subjects provided written informed consent and experimental procedures were approved by the Ethics Committee of the University of Hasselt. The study conforms to the principles stated in the Declaration of Helsinki.

### Subjects

Thirty-six subjects, 18 men and 18 women, aged 19 to 24 years (mean age±SD = 20.47±1.21) participated in this study. Handedness was assessed with the Edinburgh Handedness Inventory [23]. All participants were right-handed (mean LQ±SD = 91.16±13.32) and naïve for TMS. The resting motor threshold (rMT) ranged between 32% and 50% of the maximum stimulator output (mean rMT±SD = 39.81±4.70). Before inclusion all subjects were screened for TMS contra-indications [24]; and for pathologies associated with peripheral and/or central sensory dysfunction and for central nervous system-acting, psychotropic or antiepileptic medication intake.

### Experimental Design

Prior to the start of the experiment, subjects were asked to report their level of attention, fatigue and arousal using a visual analogue scale (VAS) score. Next, the hotspot of the first dorsal interosseous (FDI) muscle and the rMT was determined using TMS. Then, subjects moved on to a double-blind crossover procedure. Both, subjects and the experimenter applying TMS were blinded for the stimulation intensity. Subjects received two blocks of 40 consecutive TMS pulses, one block at 110% rMT and another at 120% rMT. Neither the experimenter nor the subjects received feedback (visualization of the MEPs). Subjects were instructed to keep their eyes open during the experiment. After the experiment, VAS scores were assessed again to monitor changes in attention, fatigue and arousal during the experiment, which lasted approximately 30 min.

### Transcranial Magnetic Stimulation (TMS)

Magnetic stimuli (Magstim BiStim^2^, Whitland, South West Wales, UK) were delivered using a 70-mm loop-diameter figure-of-eight coil. For each subject, an orthogonal 1×1 cm coordinate system was marked on a swimming cap with references to anatomical landmarks (left and right external auditory meatus, occiput and vertex). Then, the hotspot (scalp location resulting the highest mean MEP after five consecutive magnetic stimuli) of the relaxed FDI muscle was determined. The coil was positioned on the left hemisphere with the coil handle pointing backward and rotated 45° away from the midsagital line [11]. Next, the rMT was defined as the lowest stimulation intensity evoking MEPs with an amplitude larger than 50 µV peak-to-peak in at least five of ten consecutive trials. Finally, two blocks of 40 consecutive TMS pulses (40 pulses at 110% rMT and 40 pulses at 120% rMT) were administered in a randomized order. A two-minute break was provided between blocks. The interval between TMS stimuli was randomized (5–8 s.).

### Electromyographic Recordings (EMG)

Electromyographic signals from the FDI muscle were continuously monitored and measured using EMG (Bagnoli-16, Delsys Inc, Boston, USA). After amplification (gain = 1000), band pass filtering (4–1500 Hz) and 50/60 Hz noise elimination (Humbug, Quest Scientific, North Vancouver, Canada) the recorded EMG signals were digitized at 5000 Hz (CED Signal Version 3.03, Cambridge Electronic Design, Cambridge, UK) and were stored on a laboratory computer for offline analysis.

### Data Analysis

#### Visual Analogue Scale (VAS)

Because the Shapiro-Wilk test indicated that the data was not normally distributed, the Wilcoxon signed-rank test was used for statistical analysis of the VAS scores. The level of significance was set at p<0.05.

#### MEP data analysis

Before analysis, individual MEPs were screened for artefacts and voluntary contraction; and excluded (<1%) if the root mean square EMG exceeded 5 µV during the 50-ms period immediately preceding the onset of the TMS pulse.

**Figure 1:**
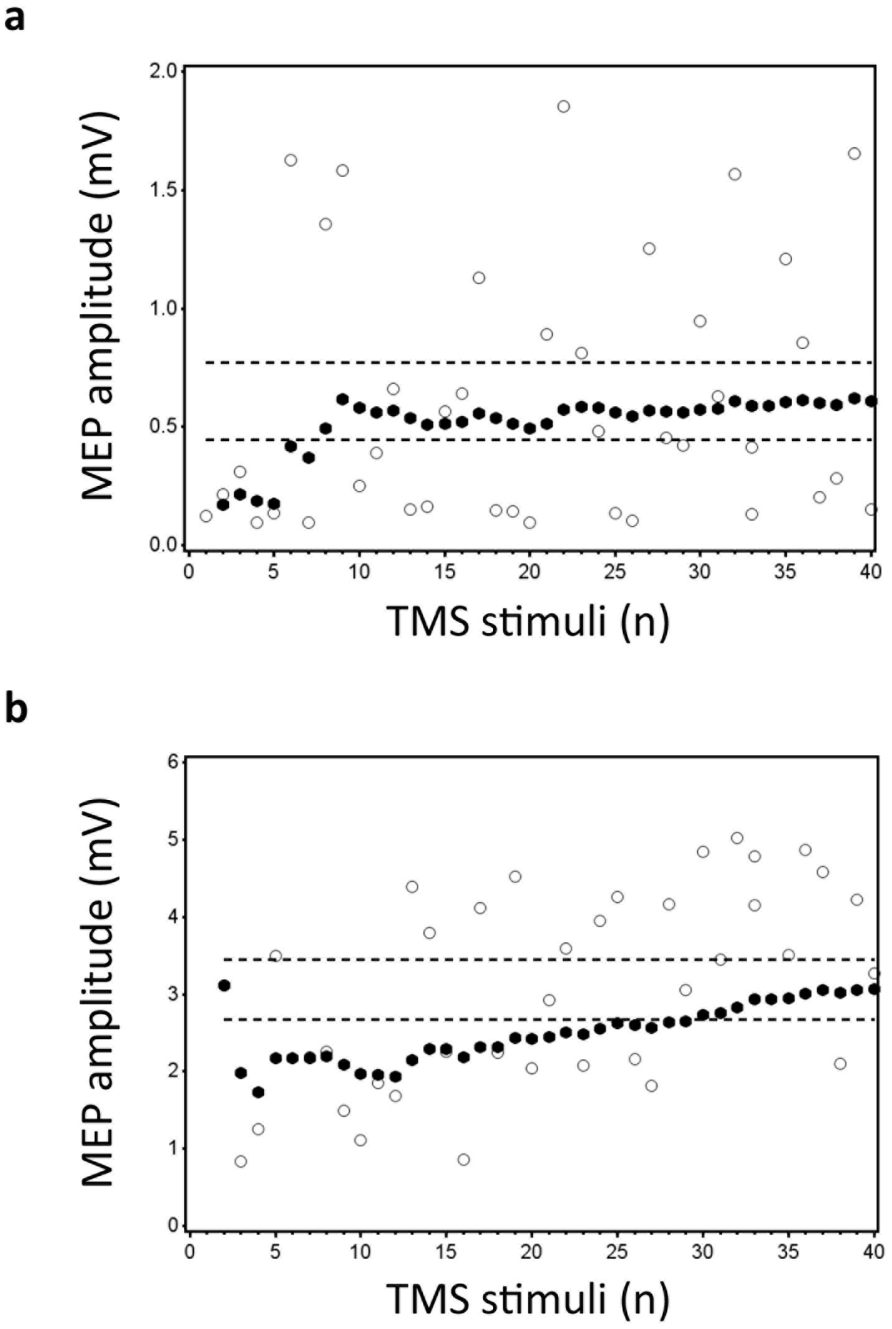
Data for subject 36 (a) and 34 (b) is illustrated. The Y-axis shows the MEP amplitude (mV), while the number of TMS stimuli (n) is shown on the X-axis. White dots represent the individual (raw) MEPs, whereas the black dots represent the average of consecutive MEPs (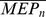). Dashed lines represent the 95% confidence interval (CI), which is based upon all 40 stimuli. The upper panel (**a**) illustrates data that was included in the statistical analysis (slope estimate: 0.007; p = 0.355). For this particular subject, 8 consecutive stimuli were sufficient to enter the CI. The lower panel (**b**) shows data that has been excluded due to a significant change in slope over time (slope estimate: 0.062; p<0.001).

In the main analysis, data of all 36 subjects were analysed. For each subject, the average MEP was calculated for subsets of consecutive stimuli: 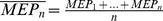, where n: 2…40. In this experiment 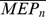 can be assumed as most accurate estimate of the true underlying MEP value. The main goal of this study was to define the number of consecutive stimuli needed for to approach 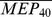. In order to evaluate the accuracy of the estimates, a 95% confidence interval (CI) was calculated using all 40 stimuli for each subject (see figure 1). Based on both the 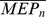
values and the CI, it is possible to determine whether 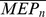 is included in CI, yielding a binary variable (0 = not included in the CI, 1 = included in the CI). The procedure described above was applied to all subjects for both stimulation intensities. Additionally, we analysed if either gender and/or stimulation intensity had an effect on the total number of stimuli required to obtain a reliable estimate of CSE. The change in attention (post TMS – pre TMS), arousal, and fatigue; and rMT were included as covariates in our model. The level of significance was set at p<0.05. In order to investigate these effects a generalized estimating equation (GEE) analysis was used (SAS v9.2). More specifically, this technique estimates the parameters of a general linear model taking into account the correlation between two measurements of the same subject with different stimulation intensities. In the current analysis a binary response variable indicates whether an estimate of the true underlying MEP value (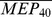), based on a certain number of repetitions, falls within the confidence interval (response = 1) or not (response = 0). This yields a vector consisting of the values 0 and 1 for each subject. Since there exists no convenient specification of the joint distribution for such a vector of binary responses, Generalized Estimating Equations (GEE) was introduced as a valuable alternative for maximum likelihood estimation [25], [26]. In more detail, within the GEE approach a marginal model is considered with a mean function of the predictor variables similar to the well-known linear models in regression and ANOVA. Furthermore, within the GEE approach this is combined with a known variance function and a working correlation matrix. The main idea is to generalize the univariate likelihood equations by means of a covariance matrix of the response vector.

Although the MEP amplitude is highly variable, no significant trend in MEP amplitude over time is expected during rest [27]. Therefore, we performed an identical supplementary analysis. However, if a significant change (slope) in CSE over time was detected (using a simple linear regression analysis) within one of the stimulation intensities, the data corresponding to this intensity was excluded from the analysis. After excluding subjects 2 [Male (M)], 11 (M), 12 (M), 16 [Female (F)], 17 (F) and 26 (F) for 110% rMT and subjects 8 (F), 10 (F), 14 (M) and 34 (F) for 120% rMT, respectively 30 (110% rMT) and 32 (120% rMT) subjects were maintained in each condition for statistical analysis (see figure 1 for an example of included/excluded data).

## Results

### Estimation of Corticospinal Excitability

Table 1 shows the probability that the average of these stimuli will be in the CI for the total sample irrespective of intensity and gender as a function of the number of consecutive stimuli. Exploratory data analysis revealed that a probability of 1 was reached only after at least 30 consecutive stimuli.

**Table 1:** Probability table.

Number of consecutive stimuli	Probability of hitting the 95% CI
2	0.39
3	0.30
4	0.32
5	0.38
6	0.38
7	0.44
8	0.50
9	0.55
10	0.58
11	0.58
12	0.65
13	0.66
14	0.65
15	0.71
16	0.78
17	0.79
18	0.82
19	0.83
20	0.86
21	0.86
22	0.86
23	0.89
24	0.90
25	0.92
26	0.99
27	0.99
28	0.99
29	0.99
30–40	1.00

The number of consecutive stimuli required as a function of the probability of hitting the 95% confidence interval (CI).

**Figure 2:**
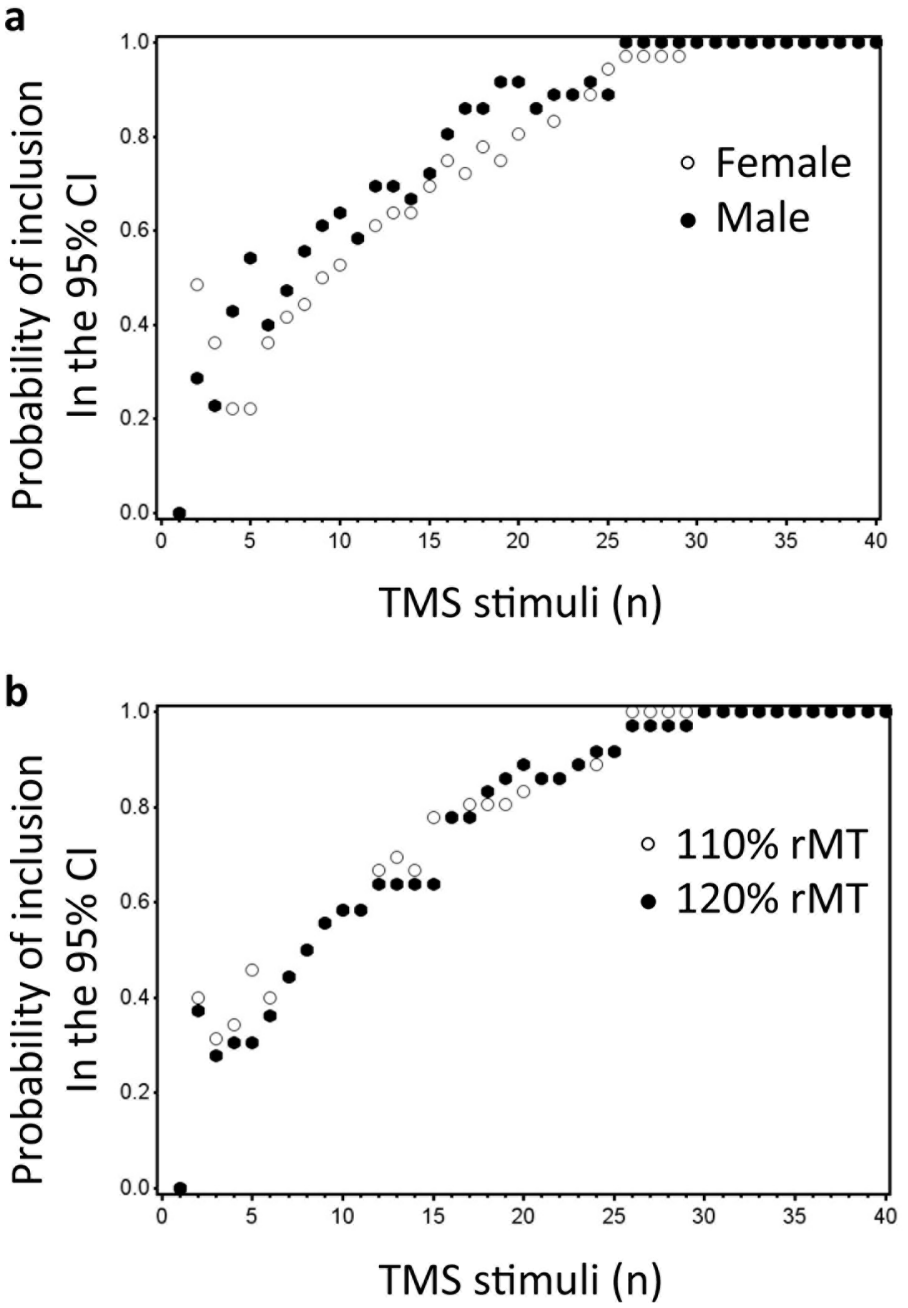
Results for gender (a) and intensity (b) based upon the raw data are illustrated. The Y-axis shows probability of inclusion in the 95% CI, while the number of TMS stimuli (n) is shown on the X-axis. The upper panel (**a**) illustrates the probability of inclusion in the 95% CI for females (white dots) and males rMT (black dots). The lower panel (**b**) illustrates the probability of inclusion in the 95% CI for the stimulation intensity of 110% rMT (white dots) and 120% rMT (black dots).

The GEE analysis (Table 2) showed only a significant effect for number of consecutive stimuli, indicating that the probability that the estimate will be in the CI increased when the number of consecutive stimuli increased (p = 0.033). No significant effects were reported for stimulation intensity or gender (all, p>0.05; see Figure 2). Explorative data analysis showed that, at least 26 consecutive stimuli were required to reach a probability of 1, when stimulating at an intensity of 110% rMT and at least 30 consecutive stimuli at 120% rMT. For females 30 consecutive stimuli were required, while for males 26 consecutive stimuli were needed to reach a probability of 1.

**Table 2:** Generalized estimating equation (GEE) analysis.

Parameter	Estimate	Z-value	p-value
Intercept	−4.7675	−3.24	**0.001**
Number of stimuli	0.2333	2.13	**0.033**
Resting motor threshold	0.0715	2.35	**0.019**
Resting motor threshold× Number of stimuli	−0.0019	−0.71	0.481

Estimates and p-values are shown for the number of consecutive stimuli, arousal, fatigue, resting motor threshold and the interaction between arousal and fatigue. P-values in bold highlight a significant effect.

### Resting Motor Threshold

The GEE analysis showed a significant effect of rMT on CSE estimates (p = 0.019). Moreover, as shown in Table 3, for subjects with a higher rMT, fewer consecutive stimuli were required to reach a stable estimate of CSE.

**Table 3:** The number of TMS stimuli required to reach a probability of 1.0 for hitting the 95% CI was estimated using the GEE analysis for different levels of resting motor threshold (rMT).

rMT(% max. stim.output)	TMS stimuli requiredfor probability = 1.0
32–34	28
35–42	27
43–47	26
47–50	25

### Visual Analogue Scale Scores

Table 4 shows the data for attention, fatigue and arousal obtained prior and after the experiment. For the entire group a significant decrease in attention (p<0.001), arousal (p<0.001) and a significant increase in fatigue (p = 0.014) were reported during the course of the experiment. Similar results were reported for males (all, p<0.05). However, for females, there was only a significant decrease in arousal (p = 0.026). Attention and fatigue did not change significantly over time in this subgroup (all, p>0.05).

**Table 4:** Visual Analogue Scales (VAS).

Parameter		PRE		POST		Z-value	p-value
		Mean	SD	Mean	SD		
	Allsubjects	6.30	(1.70)	5.31	(1.80)	−3.567	**<0.001**
Attention	Male	6.67	(1.52)	5.47	(2.03)	−3.435	**<0.001**
	Female	5.93	(1.83)	5.16	(1.58)	−1.801	0.074
	Allsubjects	5.71	(1.99)	5.24	(2.22)	−2.436	**0.014**
Fatigue	Male	6.16	(1.70)	5.23	(2.03)	−2.746	**0.004**
	Female	5.27	(2.19)	5.26	(2.46)	−0.632	0.543
	Allsubjects	3.32	(2.37)	2.23	(2.11)	−3.441	**<0.001**
Arousal	Male	2.53	(1.96)	1.76	(2.01)	−2.842	**0.003**
	Female	4.10	(2.53)	2.69	(2.15)	−2.200	**0.026**

The mean VAS score ± standard deviation (SD) is shown for attention (0 = no attention; 10 = maximal attention), fatigue (0 = no fatigue; 10 = maximal fatigue) and arousal (0 = no arousal; 10 = maximal arousal). Measurements were obtained prior (PRE) and after (POST) the experiment. Data is shown for all subjects and for males and females separately. P-values in bold highlight a significant effect between PRE and POST measurement.

In addition, the GEE analysis revealed no effect for none of these covariates on the CSE estimates (all, p>0.05), indicating that neither the change in attention, nor the change in arousal or fatigue influenced the CSE estimates.

### Supplemental Data Analysis

Similar results were obtained after exclusion of subjects who showed a significant trend in MEP amplitude over time (for details, see Supporting Information). Moreover, the GEE analysis (Table S2) revealed only significant effects for the number of pulses (p<0.001) and rMT (p = 0.005, Table S3). There were no effects for gender, attention, arousal, fatigue, or their interactions (all, p>0.05). The number of consecutive pulses required to obtain the most reliable estimate for CSE was at least 26 (Table S1).

## Discussion

The primary aim of the present study was to optimize the TMS protocol for acquiring a reliable estimate of CSE. During our measurements, we attempted to control for the factors affecting MEP variability, such as pre-stimulus contraction [1], [8], attention [9], arousal and coil orientation [11]. Our results revealed that at least 30 consecutive stimuli were required to obtain the most accurate estimate of CSE. In addition, stimulation intensity and/or gender had no significant effect on the estimation of CSE.

As the MEP amplitude is highly variable within subjects [1]–[4] several consecutive TMS stimuli were applied in most studies. Until now, no clear advice was available with respect to the amount of stimuli required to obtain reliable estimates of CSE. This basic methodological information might play a crucial role in the development of reliable TMS protocols.

Interestingly, our results revealed no significant differences between two frequently used intensities (110% and 120% rMT). As illustrated in studies measuring TMS recruitment curves [28]–[30], these intensities are well situated in the rising part of the (sigmoidal) curve, making them sensitive candidates for evaluating shifts in CSE.

In the current study no significant gender effect was reported. This finding was in line with Pitcher et al. (2003), who did not found a main-effect of gender on MEP variation when exploring TMS recruitment curve characteristics. However, they did report a significant interaction between stimulus intensity, age, rMT and gender. Moreover, females tended to have increased MEP variability than males, but age and rMT were much stronger modulators of MEP variation than gender [5]. In contrast with our results, previous findings [20], [21] indicated that MEP variation was greater in females (due to changes in ovarian steroid levels during the various stages of the menstrual cycle). Nevertheless, we should be careful with comparing our results with the gender-related differences reported in earlier studies because different TMS protocols were used focussing primarily on inhibitory networks [20], [22]. More specifically, it is thought that variability in inhibition is due to allosteric action of progesterone-derived neurosteroids on GABA_A_ receptor transmission [31], [32], indicating that ovarian hormones exert an effect on brain function. However, it might be argued that gender-related variability in specific intracortical γ-aminobutyric acid A (GABA_A_)-mediated inhibitory networks within M1 may not necessary translate into variability in the activation of the cortical network targeted by single-pulse TMS.

Our results revealed that rMT contributed significantly to the estimation of CSE. More specifically, for subjects with a higher rMT, fewer stimuli were required to reach a stable estimate of CSE. This is in line with Smith et al. (2011), indicating that subjects with high rMTs showed less MEP variability at a given stimulus intensity, as compared to subjects with low rMTs [30]. Unfortunately, the current literature offers no obvious neurophysiological explanation for this finding.

Though the current results are clear, we need to be cautious with the interpretation of our results. Firstly, we have to be careful with extrapolating our findings to other populations. Since our data was obtained in healthy young subjects, a different number of consecutive stimuli might be required when estimating CSE in elderly or neurodegenerative populations. As compared to young subjects, trial-to-trial variability was shown to be increased in elderly, specifically at low, near threshold intensities [5]. In patients suffering from neurodegenerative disease, it is reported that MEP amplitudes are often reduced or even absent [33]–[35]. Secondly, estimates of CSE can be influenced by the experimental set-up. For example, the use of navigated TMS, different coil types and shapes, EMG hardware configuration and noise elimination can affect variability and reliability of the measurements. With respect to TMS navigation, a recent study comparing non-navigated and MRI-guided navigated TMS [36] reported that the stability of MEPs increased significantly (lower MEP variability) when MRI-guided navigated TMS was used. In contrast, findings from Jung et al. (2010) revealed no significant difference in MEP variability and reproducibility between non-navigated and optically tracked TMS navigation [37]. Although we did not use MRI-guided navigated TMS in the current study, there is evidence that experimenters using non-navigated TMS can reach a performance level, which is comparable with optically tracked navigated TMS measurements as indicated by Jung et al. (2010).

With respect to reliability and accuracy of CSE, the triple-stimulation technique [38] has shown to be superior as compared to the current (conventional) technique. Nonetheless this advanced technique has also limitations, as it is more complex, only suitable for distal muscles and less comfortable for the subject. Furthermore, as the majority of the TMS studies are performed with the conventional technique, our results yield important information for designing TMS experiments using the conventional technique.

Although, we used a standardized procedure and attempted to control for attention, fatigue and arousal, these parameters can still change during the course of the experiment, as illustrated by our results. Nonetheless all subjects were comprehensively briefed with respect to the experimental procedures, the perceived changes in attention, arousal and fatigue throughout the experiment might be explained by an increased level of arousal and attention and a decrease level of fatigue prior to the experiment due to uncertainty about their first TMS experience [39]. Therefore, a familiarization session prior to the actual experiment might be recommended to minimize these effects. With respect to attention and arousal of the subjects, our results are in line with Hess et al. (1987). They reported that the threshold for excitation of the relaxed muscle showed some variation over time, but that is was not related to attention or alertness of the subject [40]. Furthermore, during TMS measurements background EMG was monitored to make sure the level of muscle relaxation was constant. With respect to fatigue in particular, previous studies reported that MEP amplitude decreased due to fatigue or to increased relaxation of the stimulated muscle [41], [42]. This might explain why we found a trend in the change of MEP amplitude over time in some subjects. However, excluding these subjects did not dramatically change our results. Furthermore, when subjects who showed a trend in MEP amplitude over time were included in the analysis, the current results showed that, overall, the application of only 4 extra pulses was sufficient to obtain a reliable estimation of CSE.

Importantly, although VAS scales indicated that subject reported changes in attention, fatigue and arousal, these perceived changes did not contribute significantly to the estimation of CSE as revealed by the GEE analysis.

In summary we can conclude that with the current TMS procedure, at least 30 consecutive stimuli are required to obtain the most reliable estimate for CSE. In addition, no significant differences were reported for gender or stimulation intensity. rMT, however, did contribute significantly to the estimation of CSE. More specifically, for subjects with a higher rMT, fewer stimuli were required. The current findings can be used to optimize the design of TMS experiments.

## Supporting Information

Table S1 **Probability table.** The number of consecutive stimuli required as a function of the probability of hitting the 95% confidence interval (CI).
(DOCX)

Table S2 **Generalized estimating equation (GEE) analysis.** Estimates and p-values are shown for the number of consecutive stimuli, arousal, fatigue, resting motor threshold and the interaction between arousal and fatigue. P-values in bold highlight a significant effect.
(DOCX)

Table S3 **The number of TMS stimuli required to reach a probability of 1.0 for hitting the 95% CI was estimated using the GEE analysis for different levels of resting motor threshold (rMT).**
(DOCX)
