# Supplementary material for: Optimization of the Transcranial Magnetic Stimulation Protocol by Defining a Reliable Estimate for Corticospinal Excitability
Source: PLoS One. 2014 Jan 24;9(1):e86380. doi: 10.1371/journal.pone.0086380 (PMC3901672; doi:10.1371/journal.pone.0086380)
Supplement: Table S1 — Probability table. The number of consecutive stimuli required as a function of the probability of hitting the 95% confidence interval (CI). (DOCX) [file pone.0086380.s001.docx]

| Number of | Probability of |
| --- | --- |
| consecutive stimuli | hitting the 95% CI |
| 2 | 0.40 |
| 3 | 0.33 |
| 4 | 0.33 |
| 5 | 0.41 |
| 6 | 0.43 |
| 7 | 0.48 |
| 8 | 0.58 |
| 9 | 0.65 |
| 10 | 0.68 |
| 11 | 0.68 |
| 12 | 0.76 |
| 13 | 0.76 |
| 14 | 0.74 |
| 15 | 0.81 |
| 16 | 0.89 |
| 17 | 0.90 |
| 18 | 0.92 |
| 19 | 0.95 |
| 20 | 0.95 |
| 21 | 0.92 |
| 22 | 0.92 |
| 23 | 0.95 |
| 24 | 0.95 |
| 25 | 0.95 |
| 26-40 | 1.00 |
|  |  |
