# Supplementary material for: Optimization of the Transcranial Magnetic Stimulation Protocol by Defining a Reliable Estimate for Corticospinal Excitability
Source: PLoS One. 2014 Jan 24;9(1):e86380. doi: 10.1371/journal.pone.0086380 (PMC3901672; doi:10.1371/journal.pone.0086380)
Supplement: Table S2 — Generalized estimating equation (GEE) analysis. Estimates and p-values are shown for the number of consecutive stimuli, arousal, fatigue, resting motor threshold and the interaction between arousal and fatigue. P-values in bold highlight a significant effect. (DOCX) [file pone.0086380.s002.docx]

| Parameter | Estimate | Z-value | p-value |
| --- | --- | --- | --- |
| Intercept | -4.7675 | 3.70 | **<0.001** |
| Number of stimuli | 0.2003 | -11.82 | **<0.001** |
| Arousal | 0.0139 | -0.23 | 0.819 |
| Fatigue | 0.0457 | -0.33 | 0.740 |
| Resting motor threshold | 0.0877 | -2.79 | **0.005** |
| Arousal x Fatigue | 0.0784 | -1.80 | 0.071 |
