# Supplementary material for: Optimization of the Transcranial Magnetic Stimulation Protocol by Defining a Reliable Estimate for Corticospinal Excitability
Source: PLoS One. 2014 Jan 24;9(1):e86380. doi: 10.1371/journal.pone.0086380 (PMC3901672; doi:10.1371/journal.pone.0086380)
Supplement: Table S3 — The number of TMS stimuli required to reach a probability of 1.0 for hitting the 95% CI was estimated using the GEE analysis for different levels of resting motor threshold (rMT). (DOCX) [file pone.0086380.s003.docx]

| rMT | TMS stimuli required |
| --- | --- |
| (% max. stim.output) | for probability = 1.0 |
| 32 - 35 | 25 |
| 36 - 40 | 22 |
| 41 - 45 | 20 |
| 46 - 50 | 20 |
|  |  |
